# A Complete Response in a Metastatic Melanoma Patient After a Single Dose of Dual Checkpoint Inhibitors Blockade Could Be Predictable: A Case Report

**DOI:** 10.7759/cureus.69301

**Published:** 2024-09-12

**Authors:** Raluca Ioana Mihaila, Adelina Silvana Gheorghe, Daniela Luminita Zob, Dana Lucia Stanculeanu

**Affiliations:** 1 Department of Oncology, University of Medicine and Pharmacy Bucharest, Bucharest, ROU; 2 Department of Medical Oncology I, Institute of Oncology "Prof. Dr. Alexandru Trestioreanu", Bucharest, ROU; 3 Department of Medical Oncology I, Institute of Oncology “Prof. Dr. Alexandru Trestioreanu”, Bucharest, ROU; 4 Department of Medical Oncology II, Institute of Oncology “Prof. Dr. Alexandru Trestioreanu", Bucharest, ROU; 5 Department of Medical Oncology, Institute of Oncology "Prof. Dr. Alexandru Trestioreanu", Bucharest, ROU

**Keywords:** complete response, immune adverse events, pd-l1, ctdna, tmb, pheriperal blood biomarkers, predictive biomarkers, immunotherapy, metastatic melanoma

## Abstract

Cutaneous malignant melanoma is one of the most aggressive forms of skin cancer and thus, a high mortality has been reported over decades. The prognosis for melanoma varies widely based on several factors, including the stage at which it is diagnosed, the location and thickness of the tumor, the patient's age and overall health, and specific genetic factors associated with melanoma. Therapeutic options include checkpoint inhibitors, regardless of V-Raf Murine Sarcoma Viral Oncogene Homolog B status (BRAF), and targeted therapy (anti-BRAF) in the adjuvant or metastatic setting. Immune checkpoint inhibitors (ICIs) have revolutionized cancer treatment but predicting which patients will benefit from these therapies remains challenging. Biomarkers like leukocytes, neutrophils, eosinophils, basophils, platelets, and other peripheral blood biomarkers have been investigated for their potential to predict responses to ICIs. Tumor mutational burden (TMB), circulating tumor DNA (ctDNA), and soluble PD-L1 (sPD-L1) have emerged as potential biomarkers for predicting responses to ICIs. Elevated baseline levels of ctDNA and elevated sPD-L1 levels have been associated with worse prognosis in melanoma patients. High TMB is often associated with better responses to ICIs in melanoma. Here we present a case from our department, of a 57-year-old patient, diagnosed in 2019 with stage IV - pT4cNx cM1 (lymph nodes metastases) and suspicion of lung metastases, BRAF wild-type right hallux malignant melanoma. Due to impressive results, first-line treatment with ICIs nivolumab and ipilimumab was the preferred treatment of choice, which showed a favorable response, with regression of oncological disease after the first cycle, and achieving complete response afterward. Unfortunately, the treatment was discontinued due to severe hepatic and pancreatic toxicity, but the favorable response to immunotherapy has been maintained for four years and is ongoing. Identifying predictive biomarkers is important to achieve the best response for the patient, with minimal adverse events, especially if long-term clinical benefit can be reached.

## Introduction

Immune checkpoint inhibitors (ICIs) have become a cornerstone in the treatment of melanoma, significantly improving patient outcomes. However, predicting which patients will benefit from ICIs remains a clinical challenge. Various hematologic parameters, including leukocytes, neutrophils, eosinophils, basophils, platelets, and other peripheral blood serum biomarkers, have been investigated for their potential to predict responses to ICIs in melanoma [1-4].

Baseline leukocytosis has been associated with systemic inflammation and poorer outcomes in some studies, suggesting an immunosuppressive tumor microenvironment. The neutrophil-to-lymphocyte ratio (NLR) is a widely studied prognostic biomarker in melanoma treated with ICIs. A high NLR is generally associated with poor prognosis and lower response rates to ICIs [1-4]. Neutrophils may contribute to an immunosuppressive environment, hindering the efficacy of ICIs. Diem et al. (2017) reported that melanoma patients with a high baseline NLR had significantly shorter overall survival (OS) when treated with ICIs compared to those with a low NLR [1]. Eosinophil counts have been associated with favorable responses to ICIs in melanoma. High baseline or early increased eosinophil counts during ICI treatment have been linked to better outcomes, potentially due to eosinophils' role in enhancing anti-tumor immunity. The role of basophils in predicting ICI response in melanoma is less well studied. Some evidence suggests that basophils may enhance anti-tumor immunity through cytokine release and interactions with other immune cells, but their predictive value for ICI response remains unclear. Elevated platelet counts have been associated with poor outcomes in melanoma patients treated with ICIs. Platelets can promote tumor growth and metastasis by shielding tumor cells from immune surveillance and aiding their adhesion to the endothelium. Ferrucci et al. (2015) demonstrated that melanoma patients with high baseline platelet counts had significantly shorter OS when treated with ICIs than those with normal platelet counts [2].

Serum biomarkers offer a non-invasive method to potentially predict response to ICIs. Lactate dehydrogenase (LDH) is an established biomarker in melanoma, often associated with tumor burden and disease progression. Elevated baseline LDH levels have been linked to decreased OS in melanoma patients treated with ICIs. Elevated C-reactive protein (CRP) levels, an acute-phase protein that reflects systemic inflammation, have been associated with poorer prognosis in melanoma patients treated with ICIs, indicating a potential immunosuppressive environment. A study by Lauwyck J, et al. concluded that high baseline CRP levels were predictive of shorter OS in melanoma patients undergoing ICI therapy [3]. Interleukin-6 (IL-6) is a pro-inflammatory cytokine involved in the immune response, and high serum IL-6 levels have been linked to poor outcomes in melanoma patients treated with ICIs, likely due to its role in promoting an immunosuppressive tumor microenvironment. A study by Laino AS, et al. showed that elevated IL-6 levels were predictive of worse progression-free survival (PFS) and OS in melanoma patients treated with ICIs [4]. Also, serum levels of certain metabolites such as kynurenine and tryptophan, involved in the indoleamine 2,3-dioxygenase pathway, have been linked to ICI outcomes.

Combining hematological parameters with other biomarkers, such as tumor mutational burden (TMB), programmed death-ligand 1 (PD-L1) expression, and specific genetic mutations, may enhance the predictive accuracy of ICI responses. A multifactorial approach may provide a more comprehensive assessment of the tumor microenvironment and the patient's immune status, improving the prediction of ICI efficacy. Soluble PD-L1 (sPD-L1) is the circulating form of the PD-L1 protein, which can bind to programmed death (PD-1) receptors and inhibit immune responses, and elevated sPD-L1 levels have been correlated with poor outcomes in melanoma patients treated with ICIs. Zhou et al. (2017) reported that high sPD-L1 levels were associated with shorter PFS and OS in melanoma patients receiving anti-PD-1 therapy [5].

TMB represents the total number of mutations per megabase of tumor deoxyribonucleic acid (DNA) molecule and has emerged as a potential biomarker for predicting responses to ICIs. High TMB is often associated with better responses to ICIs in melanoma [6]. This correlation is due to the generation of more neoantigens from the higher mutational load, which enhances the immune system's ability to recognize and attack cancer cells. The underlying mechanism by which high TMB enhances ICI response involves the generation of neoantigens, which are novel peptides presented on the tumor cell surface due to somatic mutations. These neoantigens make the tumor cells more recognizable to the immune system, particularly to cytotoxic T lymphocytes, thereby improving the efficacy of ICIs [6]. Melanoma, characterized by a high mutational burden due to ultraviolet (UV) light-induced DNA damage, is particularly suited for TMB-based predictions. Combining TMB with other biomarkers like PD-L1 expression, microsatellite instability (MSI), and gene expression profiles may improve predictive accuracy. Not all patients with high TMB respond to ICIs, indicating the presence of other factors influencing treatment efficacy. Additionally, some patients with low TMB may still benefit from ICIs, suggesting that TMB should be part of a multifactorial approach rather than a standalone predictor [6].

Circulating tumor DNA (ctDNA), which consists of fragments of DNA shed by tumor cells into the bloodstream, offers a non-invasive method to monitor tumor dynamics and predict treatment responses. Elevated baseline levels of ctDNA have been associated with tumor burden and worse prognosis in melanoma patients. Studies have shown that high baseline ctDNA levels correlate with poor response to ICIs. Lee et al. (2017) demonstrated that melanoma patients with high baseline ctDNA had shorter PFS and OS when treated with ICIs [7]. Monitoring dynamic changes in ctDNA levels during treatment provides insights into treatment efficacy. Decreasing ctDNA levels during ICI therapy are generally associated with favorable responses and better outcomes. Conversely, increasing or stable ctDNA levels often indicate disease progression or lack of response. Gray et al. (2015) found that melanoma patients with a significant reduction in ctDNA levels within the first few weeks of ICI therapy had improved PFS and OS compared to those without such reductions [8]. ctDNA can be used to estimate TMB, which is a predictive biomarker for ICI response. Gandara et al. (2018) reported that ctDNA-derived TMB was predictive of ICI efficacy in non-small-cell lung cancer, suggesting potential applicability in melanoma [9]. ctDNA analysis can identify specific genetic mutations associated with ICI response. Mutations in genes such as BRAF, NRAS, and TP53 can influence treatment outcomes. Anagnostou et al. (2020) demonstrated that melanoma patients with ctDNA-detected BRAF mutations responded better to ICIs than those without these mutations [10]. ctDNA is valuable for detecting minimal residual disease and early recurrence. Persistent or rising ctDNA levels after the initial response can indicate impending relapse. A study by Cabel et al. (2017) highlighted that melanoma patients with detectable ctDNA post-treatment were more likely to experience recurrence, emphasizing the utility of ctDNA in long-term monitoring [11].

## Case presentation

**Figure 1:**
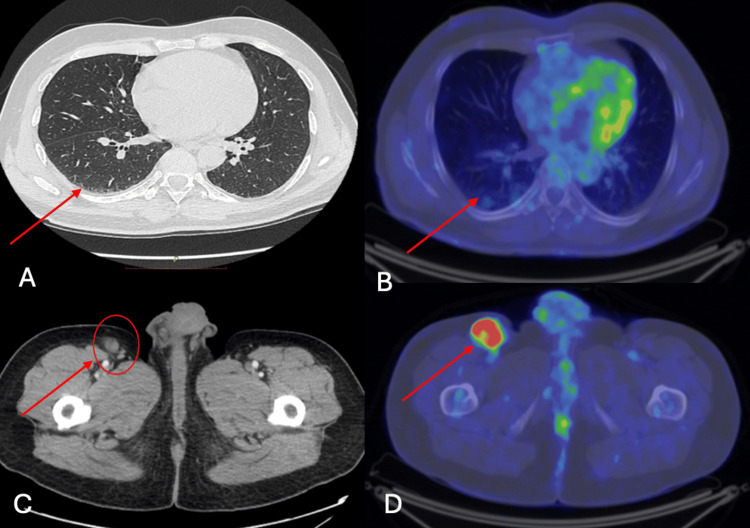
Baseline CT scan and PET-CT A: CT scan - rare lung micronodules - to be monitored. B: PET-CT - metabolically inactive bilateral lung nodules. C: CT scan - 47/33 mm right inguinal lymphadenopathy. D: PET-CT - confirmed metabolically active right inguinal lymphadenopathy.

Here we present an interesting case from our department, of a 57-year-old patient, diagnosed in 2019 with stage IV pT4cNx cM1 (lymph nodes metastases) and suspicion of lung metastases, proto-oncogene B-Raf (BRAF) wild-type right hallux malignant melanoma that achieved partial remission initially, afterward complete response with dual ICIs nivolumab (NIVO) and ipilimumab (IPI). The patient, with no family or personal medical history, presented himself for a consult in July 2019 at the Oncological Institute “Professor Dr. Alexandru Trestioreanu” from Bucharest in July 2019 with a right hallux tumor. The examination did not reveal other signs and symptoms, except for the primary tumor formation and ipsilateral inguinal adenopathy in the femoral area, which appeared approximately six months ago, with progressive growth and associated mild algid syndrome. The initial staging CT scan revealed a 47/33 mm right inguinal lymphadenopathy and rare lung micronodules to be monitored due to diagnosis; no cerebral, hepatic, and bone metastases were identified. The multidisciplinary team (MDT), which included oncology, radiotherapy, surgery, pathology, and radiology, decided that PET-CT should be performed and the results confirmed metabolically active right inguinal lymphadenopathy, metabolically inactive bilateral lung nodules, but suspicious on CT scan for distant metastases. Thus, it was decided in the MDT meeting that a diagnostic excision was the first step in the multimodal treatment approach (Figure 1).

The histopathological examination of the right hallux tumor formation identified a tumor formation with a diameter of 4 cm, with a piece of skin excision showing an ulcerated malignant cell proliferation, consisting of large cells, with an epithelioid appearance, with prominent nuclei and increased mitotic activity (32M/mmp). The neoplastic cells were arranged in trabecular patterns and islands, focally showing lentiginous areas at the levels of the dermo-epidermal junction. Aspects of perineural and lymph vascular invasion (PNI, LVI) were present. Proliferation extends in depth over a distance of at least 13 mm, on the examined fragments and corresponds to a Clark V index. A "non-brisk" peritumoral inflammatory infiltrate was observed. The presence of melanic pigment was not observed. The piece of skin excision showed aspects of extensive melanoma on the surface and in the depth of the subcutaneous adipose tissue a melanocytic proliferation with characters similar to those described before. At the part of the hallux amputation, which showed a malignant cell proliferation on the surface, characteristics similar to those described previously were observed, with a diffuse infiltration of the superficial bone plane. Tumor emboli and LVI and PNI were present at this level. Conclusions of the final report stated that histopathological aspects are suggestive of an ulcerated malignant melanoma, developed extensively on the surface, with a vertical growth phase, presenting a Breslow index of at least 13 mm, Clark V index, pT4bNxMxLVI, and PNI. Immunohistochemistry markers tested and identified are detailed in Table 1. No lymphatic emboli were evident on the examined sections and the immunohistochemistry tests support the diagnosis of malignant melanoma with surface extension and vertical growth phase with a Breslow index of 18 mm and Clark V index. 

**Table 1:** Immunohistochemistry markers tested and identified on the right hallux primary tumor

Immunohistochemistry markers	Result
Melanoma diagnostic IHC (HMB-45 + MART-1, Melan A [A103] + tyrosinase [T311])	Diffuse positive in tumor cells
pS100	Diffuse positive in tumor cells
Ki67	Positive with a nuclear index of 80%
CD34	Positive in endothelial cells marking the vascular architecture at the level of the invasion front
SOX10	Diffuse positive in tumor cells
D2-40	Positive in endothelial lymphatic cells

The histopathological and immunohistochemistry report confirmed the diagnosis of stage IV malignant melanoma, pT4bNxMx, developed on the surface, with vertical growth phase, Breslow 18 mm, Clark V, ki 67 80%, and LVI and PNI. Additional immunohistochemistry and molecular biomarkers testing was indicated (as per Table 2). Determination of BRAF mutation in this case indicated a BRAF wild-type patient. We concluded that the final diagnosis was a BRAF wild-type stage IV - right hallux cutaneous malignant melanoma - pT4NxM1, with lymph node metastasis and a suspicion of lung metastasis. Additionally, the report could determine on the immunohistochemistry tests that the tumor had microsatellite stable status (MSS). Although it is not mandatory or determined routinely, the expression of PD-L1 was evaluated by immunohistochemistry and the patient-analyzed PD-L1 status suggested an overexpression of PD-L1 (PD-L1 tumor proportion score > 51%). Any advanced tests (other molecular targets - RAS, KIT, NTRK, ALK, next-generation sequencing (NGS), uncommon genetic drivers detected by NGS panel, and evaluation of TMB) are not reimbursed by the National Insurance in Romania.

**Table 2:** Additional immunohistochemistry and molecular biomarkers tests TPS, tumor proportion score; NGS, next-generation sequencing; TMB, tumor mutational burden; PD-L1, programmed death-ligand 1.

Biomarkers	Test	Result	Interpretation
MMR proteins, including MLH1, PMS2, MSH2, and MSH6	Immunohistochemistry	Conserved nuclear expression in tumor proliferation	Microsatellite stable status
BRAF V600 gene	Real-time polymerase chain reaction	No BRAF mutation	BRAF wild type
PDL1	Ventana BenchMark GX automatic system immunohistochemistry OptiView DAB detection kit and the VENTANA PD-L1 antibody (SP263)	TPS =>51%	High PD-L1
RAS, KIT, NTRK, ALK	Not determined (not reimbursed)		
NGS			
TMB			

The patient's performance status was ECOG 0, with normal blood tests, normal hematologic parameters (including leukocytes, neutrophils, eosinophils, basophils, and platelets), an increased LDH of 330 U/L (1.5× upper normal range), normal CRP (0.2 mg/dL), and IL-6 (3 pg/mL); the patient had a negative screening for viral or bacterial infections and a normal test result for the evaluation of thyroid function (both mandatory in our National Insurance reimbursement protocol). 

The MDT therapeutic decision for this patient, hence the recommendations from NCCN guidelines in 2019, after the patient signed the informed consent, was to initiate systemic treatment with dual immunotherapy with NIVO 1 mg/kg plus IPI 3 mg/kg, for four induction cycles, every three weeks, with NIVO maintenance therapy after four cycles and imagistic revaluation and rediscussing the case in MDT meeting. The first dose was well tolerated, with no acute adverse events. But when the patient returned for the second cycle at three weeks as per protocol, his performance status was modified - ECOG 1, he presented with mild jaundice, pain in the right hypochondrium, painful abdomen and epigastric region at clinical examination, and loss of appetite. Blood tests (as per Table 3) revealed hepatic cytolysis and hepatic cholestasis, with increased direct bilirubin, amylase and lipase, and LDH. No other blood test was modified and a full viral screening for hepatitis was redone to rule out viral etiology for acute hepatic dysfunction. 

**Table 3:** Patient’s modified blood test parameters after second cycle of dual ICIs GOT, glutamic-oxaloacetic transaminase; GPT, glutamate-pyruvate transaminase; GGT, gamma-glutamyl transferase; ICI, immune checkpoint inhibitor; LDH, lactate dehydrogenase.

Test	Value	Normal range	Interpretation
GOT	519 U/L	5-34 U/L	Hepatic cytolysis
GPT	1074 U/L	6-55 U/L	Hepatic cytolysis
GGT	369 U/L	12-64 U/L	Hepatic cytolysis
Total bilirubin	6.98 mg/dL	0.1-1.2 mg/dL	Hepatic cholestasis
Direct bilirubin	5.21 mg/dL	0.0-0.5 mg/dL	Hepatic cholestasis
Lipase	935 U/L	8-78 U/L	Pancreatitis
Amylase	419 U/L	25-125 U/L	Pancreatitis
LDH	372 U/L	125-220 U/L	Non-specific

**Figure 2:**
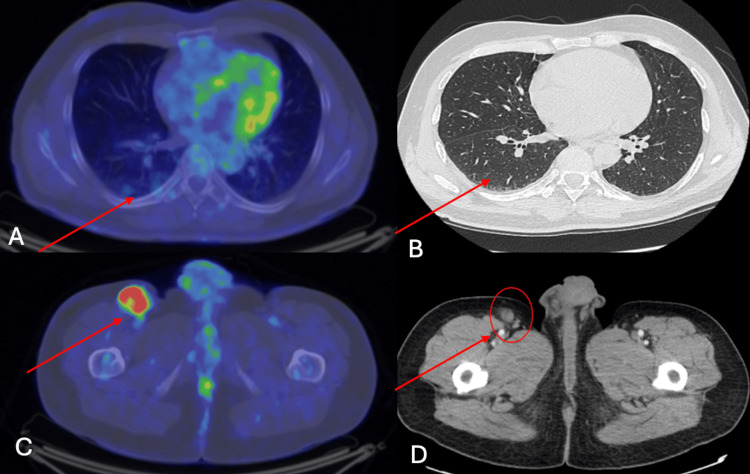
PET-CT at baseline and CT scan reevaluation after ICIs toxicity A: PET-CT baseline - metabolically inactive bilateral lung nodules. B: CT scan - rare lung micronodules - to be monitored. C: PET-CT baseline - confirmed metabolically active right inguinal lymphadenopathy. D: CT scan -  24/17 mm vs 47/33 mm (a decrease of 48.5% of the right inguinal lymphadenopathy).

We concluded that it was a severe adverse hepatic and pancreatic toxicity (as per CTCAE 2017 vs5 criteria) due to treatment and permanently discontinued immunotherapy, and reevaluated the patient in the gastroenterology department, with full imagistic workup to exclude furthermore pancreatic complications. The patient was admitted and evaluated in MDT (oncology, radiotherapy, oncological surgery, and gastroenterology) and recommendations (as per NCCN Immune Checkpoint Inhibitor-Related Toxicities) included abdominal-pelvic CT scan, methylprednisolone 1 mg/kg/day, intravenous hydration, prophylactic antibiotics, hepatoprotective treatment, pancreatic enzymes, specific diet due to acute pancreatitis, with monitoring of biological parameters. Abdominal and pelvic CT scan indicated homogeneous opaque pancreatic parenchyma (Balthazar score = 0) and oncological disease regression according to RECIST 1.1 (currently 24/17 mm vs 47/33 mm - a decrease of 48.5%) of right inguinal lymphadenopathy. Fortunately, the liver and pancreatic dysfunction remitted with specific treatment, with the patient having normal blood tests and performance status after two weeks. Even though after only one dual ICI treatment with NIVO and IPI the patient had a very good early partial response, the immunotherapy was permanently discontinued (Figure 2). 

After one month, the patient's status was reassessed with normal performance status and normal blood tests (including CRP, IL-6, and LDH), and in the MDT meeting the next step decided regarding the multimodal treatment approach was local radiotherapy for the remaining right inguinal lymphadenopathy to obtain a local control of the disease. External radiotherapy with photons using the 3D-CRT (three-dimensional conformal radiation therapy) technique up to DT = 39 Gy/target volume of the right inguinal adenopathy and the external iliac ganglia and right inguinal groups (3 Gy/fr, 1 fr/day, 13 fractions) was delivered, with good tolerance and no adverse events. Follow-up three months after radiotherapy indicated the normal clinical and biological status of the patient and a CT scan was performed to assess the response and establish the next step regarding the treatment. CT scan revealed stable non-specific lung micronodules, no brain, hepatic, and bone metastases, and right inguinal lymphadenopathy in dimensional regression 17/11 mm vs 34/17 mm (a persisting ongoing response). The MDT team decided that active surveillance was the best option for this long-term ongoing response of the patient. Active surveillance included follow-up at every three months, with clinical and biological examination, dermatology consult, imagistic reevaluation - CT scan every six months or indicated. 

Follow-up was performed from January 2020 until the present, with no modifications regarding the clinical or biological status of the patient. CT scans performed indicated a slow, but constant decrease in the right inguinal lymphadenopathy as per Table 4, demonstrating that the patient is a long-term complete responder, with no evidence of the disease for three years, after discontinuing dual ICI treatment for adverse events and fully recovered organ dysfunctions. Due to toxicities that emerged during treatment and the risk involving reinitiating immunotherapy at disease progression, the patient was recommended to determine an NGS panel from the tumor, but since it is still not reimbursed in our country, the patient could not afford the test. 

**Table 4:** CT scan response reevaluation (2019-2024) NIVO, nivolumab; IPI, ipilimumab.

Date	CT scan result
06.2019 (baseline)	Right inguinal lymphadenopathy 47/33 mm
10.2019 (after C1 NIVO+IPI)	Right inguinal lymphadenopathy 34/17 mm
01.2020	Right inguinal lymphadenopathy 17/11 mm (3 months from treatment discontinuation)
06.2020	Right inguinal lymphadenopathy 9/10 mm (6 months from treatment discontinuation)
2020-2024	No evidence of disease

## Discussion

The current literature suggests that various hematologic parameters, including leukocytes, neutrophils, eosinophils, basophils, and platelets, have potential as biomarkers for predicting responses to ICIs in melanoma. High NLR and platelet counts are generally associated with poorer outcomes, while elevated eosinophil counts may predict better responses. However, the predictive value of these parameters can vary across different studies and patient populations. At baseline, the patient did not have any predictor hematological or serum factors for potential poor prognosis except LDH, with values just above the normal upper range. As for the predictive role of increased LDH, there is no strong evidence supporting it. Data from different studies support the potential benefit of using several novel serum biomarkers in predicting ICI response in melanoma. Elevated levels of LDH, CRP, sPD-L1, IL-6, and NLR have been associated with poorer outcomes, while specific serum metabolite profiles may offer additional predictive value [12-14]. However, variability in study designs, patient populations, and biomarker assessment methods highlights the need for further research to validate these findings and establish standardized protocols. 

The current literature underscores the potential of TMB as a robust predictive biomarker for ICI efficacy in melanoma. High TMB correlates with improved outcomes due to the increased neoantigen load, enhancing the immune system's ability to target tumor cells. However, the variability in response and the need for standardization in TMB assessment highlight the necessity for further research and the combination of TMB with other biomarkers. In this particular case, it would have been very interesting to reveal that the TMB status was due to the extent of the tumor response after only one cycle of ICIs. Comprehensive profiling of TMB in this melanoma patient could have revealed not only the possible explanation for this kind of early and ongoing response but also other molecular targets, taking into account that due to severe adverse events, ICI treatment was permanently discontinued. In a recent publication, Ning B et al. revealed that patients with high TMB presented significantly improved OS (HR = 0.49, 95% CI: 0.33, 0.73; p = 0.001) and PFS (HR = 0.47, 95% CI: 0.33, 0.68; p < 0.001) compared to patients with low TMB. This association was very good in the patients treated with monotherapy, but not in the patients treated with a dual ICI combination [15]. CheckMate 066 and 067 phase III clinical trials evaluating ICIs revealed that patients receiving the anti-PD-1 inhibitor NIVO alone or in combination with the anti-CTLA-4 inhibitor IPI or IPI alone have a longer survival benefit if the TMB score was high. TMB holds significant promise as a predictive biomarker for ICI response in melanoma, offering a pathway to more personalized and effective immunotherapy [16,17]. The lack of standardization in TMB assessment methods and cutoffs poses a challenge to its widespread adoption. Establishing consistent methodologies and thresholds is crucial for integrating TMB into clinical practice. Future studies should focus on standardizing TMB measurement methods, defining optimal cutoffs, and exploring the integration of TMB with other predictive markers to refine patient selection for ICIs.

ctDNA offers a non-invasive, real-time assessment of tumor genetics and dynamics, making it a practical tool for personalized medicine. Challenges include standardizing ctDNA measurement techniques and establishing validated thresholds for clinical decision-making. The current literature underscores the potential of ctDNA as a predictive biomarker for ICI response in melanoma [18]. The analysis of quantitative ctDNA changes during systemic treatment could be a valid option for response evaluation criteria, and in this case, the assessment of ctDNA would have given the opportunity to asses the extent of the partial/complete response. But more importantly, it could be a very important tool in the follow-up of the patient with a complete response on standard CT scan/PET-CT after four years. Unfortunately, neither TMB nor ctDNA could be determined due to financial restrictions (not reimbursed by the National Health Insurance).

PD-L1 expression on tumor cells has been one of the most extensively studied biomarkers for predicting response to ICIs. High PD-L1 expression correlates with better responses to immunotherapy in various cancers, including melanoma. However, PD-L1 testing is not universally predictive and may not fully capture the complex tumor-immune microenvironment. Studies have shown a correlation between PD-L1 expression levels and the efficacy of ICIs; higher PD-L1 expression in tumors tends to respond better to ICIs. As mentioned above, although it was not mandatory or determined routinely, the expression of programmed PD-L1 was evaluated by immunohistochemistry, and the patient-analyzed PD-L1 expression by immunohistochemistry analysis (Ventana SP263; Ventana Medical Systems, Inc., Tucson, AZ) was high positive (≥50% proportion of positive staining of at least 1+ intensity). In the publication in the European Journal of Cancer, Ellebaek E, et al. emphasized that PD-L1 ≥1% was an independent positive prognostic biomarker for survival in the overall cohort (MSS: HR: 0.66, CI: 0.52, 0.83, p < 0.001), supporting previous exploratory analyses of Checkmate-067, highlighting that improved clinical outcomes with combination therapy are not established in unselected patients with high (≥1%) tumor PD-L1 expression [19]. Although is not universally predictive, this could be a valid hypothesis for the achievement of a durable complete response in our patient case. An important observation regarding the histopathological report of the patient is the "non-brisk" peritumoral inflammatory infiltrate that was observed. At the time of diagnosis, in our pathology department, tumor-infiltrating lymphocytes were not determined and could have been an interesting possible predictive biomarker to investigate. 

Most immune-related adverse events (irAEs) can be effectively managed with strategies for careful monitoring and proactive therapy management, with the goal of optimizing treatment duration to prolong patient survival and maximize quality of life (QoL); sometimes no predictor factors can be identified to assess the possibility of these adverse events. Data from the literature support the possible association of irAEs with a favorable treatment response. In different retrospective studies, authors showed that there was no difference in PFS between patients without irAEs and in patients who discontinued treatment due to severe irAEs; there was a durable clinical benefit for those who achieved partial or complete response [20]. In our patient’s case, the severe irAEs were associated with an extremely favorable therapy outcome of dual ICI - NIVO and IPI - reflected by ongoing maintained complete response for almost five years. Most importantly, the most significant role of possible predictive biomarkers is to identify the best treatment option, preventing the occurrence of severe adverse reactions. 

## Conclusions

Hematologic parameters offer a promising avenue for predicting responses to ICIs in melanoma. NLR, eosinophil counts, TMB, MSI, and ctDNA have shown potential as predictive biomarkers. Combining these parameters with other biomarkers could enhance the accuracy of response prediction, leading to more personalized and effective melanoma treatments, with minimal adverse events and a good QoL. ICIs have significantly improved the prognosis for patients with melanoma. Despite this progress, predicting which patients will benefit from ICIs remains a challenge. Novel serum biomarkers, including LDH, CRP, sPD-L1, and IL-6, and metabolite signatures like NGS hold promise in predicting responses to ICIs in melanoma. These biomarkers could facilitate more personalized and effective immunotherapy by identifying patients most likely to benefit from treatment. Further research and standardization are essential to integrate these biomarkers into clinical practice.
